# HER2/neu as a Signaling and Therapeutic Marker in Uterine Serous Carcinoma

**DOI:** 10.3390/cells14161282

**Published:** 2025-08-19

**Authors:** Victoria M. Ettorre, Luca Palmieri, Valentino Clemente, Alessandro D. Santin

**Affiliations:** 1 Department of Obstetrics, Gynecology, and Reproductive Sciences, Yale University School of Medicine, New Haven, CT 06520, USA; victoria.ettorre@yale.edu (V.M.E.); luca.palmieri02@icatt.it (L.P.); 2 Gynecologic Oncology Unit, Department of Woman and Child Health and Public Health, Fondazione Policlinico Universitario A. Gemelli IRCCS, Università Cattolica del Sacro Cuore, 00168 Rome, Italy; 3 Department of Internal Medicine, Albert Einstein College of Medicine at Montefiore Medical Center, Bronx, NY 10467, USA; vclemente@montefiore.org

**Keywords:** HER2/neu, trastuzumab, pertuzumab, antibody-drug conjugates, T-DM1, T-DXd, uterine serous carcinoma

## Abstract

Research into aggressive gynecologic cancers such as uterine serous carcinoma (USC) has recently evolved from chemotherapy to the development of drugs targeting specific biomarkers differentially expressed/active in tumor cells. One such target is HER2/neu, which plays an important role in the coordination of cell growth and differentiation. Importantly, when overexpressed and/or amplified in tumor cells, the downstream tyrosine kinase of HER2/neu becomes constitutively activated, causing dysregulated gene transcription. In breast cancer patients, HER2/neu has been successfully utilized for many years as a target for multiple monoclonal antibodies and more recently antibody–drug conjugates (ADCs). Use in gynecologic malignancies has been slower, however, due to recently identified unique characteristics of HER2/neu protein expression and gene amplification in biologically aggressive tumors such as USC including its major heterogeneity and lack of apical staining when compared to breast cancer. Accordingly, the use of optimal testing algorithms for HER2/neu status in patients with USC may have important implications for the development of novel, effective, and targeted treatment modalities against this lethal variant of endometrial cancer. In this review, we discuss HER2/neu gene expression in USC, evaluate the efficacy of HER2/neu-directed therapies in both preclinical and clinical settings, and discuss possible mechanisms of resistance to HER2/neu targeting agents.

## 1. Introduction

Endometrial cancer represents the most common gynecologic malignancy in the United States, with about 70,000 new cases projected to be diagnosed in 2025 [1]. Uterine serous carcinoma (USC) is a subset of endometrial cancer that is extremely rare, representing 10% of all endometrial cancers with poor prognosis and a higher risk of relapse regardless of stage at diagnosis [2]. Consequently, even though this represents a small subset of endometrial cancer diagnoses, uterine serous carcinoma accounts for a large proportion of uterine cancer-related deaths at 39% [3]. Current standard of care treatment for USC includes chemotherapy with carboplatin and paclitaxel, as well as cytoreductive surgery and vaginal brachytherapy [4]. Given the high propensity for recurrence, targeted treatment options are desired in recurrent/metastatic disease states.

Through the research performed by The Cancer Genome Atlas Network (TCGA), the ProMisE molecular classification system has been adapted favorably over previous type 1 and type 2 classification schema for endometrial cancer. Through this classification we and others have reported that USC has a higher proportion of ERBB2 amplifications (i.e., 27–44%) compared to other endometrial cancer subtypes [5,6]. This provides us with a targetable biomarker for advanced/recurrent USC cases with the use of trastuzumab and pertuzumab, similarly to HER2-positive breast cancers. Additionally, antibody–drug conjugates (ADCs) provide targeted delivery of cytotoxic chemotherapy to biomarker-expressing cells, while avoiding off target effects to non-expressing cells. This has been utilized in the development of trastuzumab emastine (T-DM1) and most recently trastuzumab deruxtecan (T-DXd), an ADC targeting HER2 with agnostic US Food and Drug Administration (FDA) approval in HER2 expressing solid tumors [7].

Adequate evaluation of USC pathologic tissue is required to determine who is a candidate for HER2-targeted treatment. Pathologic scoring for HER2 is universally performed by immunohistochemistry (IHC); however, IHC staining patterns are dependent on tissue type, which ultimately may influence scoring algorithms. Additionally, these targeted novel treatments are subject to the development of resistance, which may alter the time in which they are utilized over other treatment options. In this review article, we will focus on HER2′s mechanism of action, its potential utilization for targeted cancer treatment, and mechanisms for resistance to targeted treatments in USC.

## 2. HER2 Mechanism of Action and Expression

### 2.1. The HER2 Pathway

HER2 (ERBB2) is a member of the epidermal growth factor receptor (EGFR) family of tyrosine kinases that serves as membrane receptors for epidermal growth factors (EGFs) [8]. Other members of this family are HER1, HER3, and HER4 [8]. While these three function in response to autocrine or paracrine signaling via epidermal growth factors (i.e., EGFs, transforming growth factor alpha, betacellulin, and neuregulins), HER2 is the only one that has no associated ligand [8].

Each member of the EGFR family is constituted by an extracellular domain, a lipophilic transmembrane domain, and the intracellular domain that is responsible for the tyrosine kinase activity [9]. Upon binding with their ligands, the EGFRs undergo conformational changes that expose the active site of the tyrosine kinase domain and induce dimerization with the same or other members of the family [10]. However, the intracellular activity of HER3 is mostly passive, and HER2 participates in signal transduction without binding to an extracellular ligand [8,11,12]. In fact, HER2 takes a critical role in EGFR-mediated signal transduction by serving as a hub for all of the other EGFR-mediated pathways [8,11,13].

Upon formation of dimers, the EGFRs undergo transphosphorylation of the tyrosine residues exposed on the respective intracellular domains [14]. Since these tyrosine residues will in turn serve as an active site to bind intracellular signaling molecules, this key event allows for the downstream activation of intracellular pathways [8,15]. In this setting, the other EGFRs seem to have the highest affinity for HER2 as a counterpart for dimerization [8,13,16,17,18]. Furthermore, HER2 has potent tyrosine kinase activity, which accounts for the enhanced signal mediated by heterodimers which include HER2 as compared to homo- or heterodimers that do not. One of the most canonical examples is represented by the homodimer HER2/HER3 where HER3 lacks intracellular catalytic activity, which is then entirely supported by HER2 [19]. Despite this asymmetry, HER2/HER3 are often considered to have the strongest activity among the possible EGFRs dimeric combinations [20,21]. Similarly, the activity of HER1 is significantly enhanced by forming heterodimers with HER2, rather than homodimers [11].

HER2 participates in activating a plethora of downstream pathways, the most important ones being the RAS/RAF/MEK/ERK and the phosphatidylinositol-3-OH/protein kinase B/mammalian target of rapamycin (PI3K/AKT/mTOR) pathways [8,22]. Other frequently activated pathways are the STATs and the SRC pathways, but HER2 has also been shown to participate in or crosstalk with many other pro-tumorigenic pathways like WNT/beta-catenin, NF-kB, JNK, or the estrogen pathways [12,23]. Regardless of the specific pathway, once the signal reaches the nucleus, all these signals tend to converge into proliferative, anti-apoptotic, and overall pro-tumorigenic stimuli at the cellular level. For instance, while the EGFRs have been shown to be crucial for embryonal development of multiple organs [19], HER2 mutations or amplifications have a strong relationship with transformation of normal cells into cancer cells [24]. The murine analogue of HER2 (neu) has been identified as an oncogene over 40 years ago [25,26,27]. Its role in oncogenesis became obvious after the expression of mutant variants of neu with increased activity in mice lead to the establishment of several models of HER2-induced breast cancer [28,29,30], as well as other cancers [31,32]. Similarly, HER2 overexpression has been shown to induce transformation in normal cells [33,34,35]. Its tumorigenic action has been associated more frequently to its overexpression, rather than its rare mutations [36,37].

**Figure 1:**
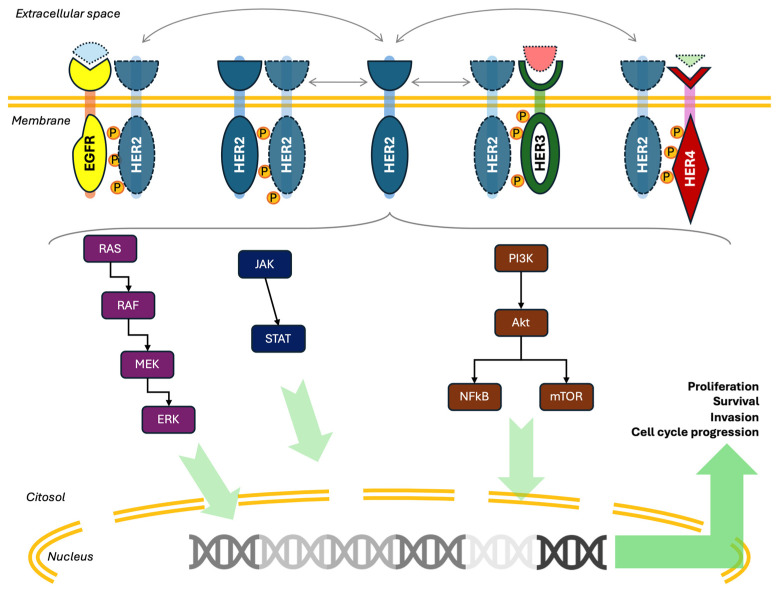
HER2 signaling diagram. HER2 is at the center of EGFR signaling at the cell membrane level. Formation of homodimers or heterodimers with the other members of the EGFR family allows for transphosphorylation of the intracellular domains and signal transduction to the cytosol. The most studied pathways downstream of HER2 are the RAS/RAF/MEK/ERK and the PI3K/AKT/mTOR pathways. Once the signal reaches the nucleus, it is eventually translated into increased proliferation, survival, and invasion.

Taken together, HER2 represents perhaps the main hub of the EGFR-mediated signaling at the cell membrane level and often acts as a bottleneck toward the downstream cascades, eventually serving as a potent promoter of cellular proliferation and tumorigenesis. A depiction of the HER2 signaling pathway is presented in Figure 1.

### 2.2. HER2 Expression in Cancer

Based on its pivotal role in EGFR signaling, HER2 has been at the center of cancer biology research for the last 30 years. Its functions have been shown to be crucial for the survival of certain tumors, and HER2-targeting molecules have been developed and are now part of standard clinical practice [12,22,24]. Historically, breast cancer was the first tumor to be associated with HER2 dependency, to the point that it is now routinely classified based on its expression [38]. The first clinically validated algorithm to access HER2 expression in pathologic specimens was developed in breast cancer, utilizing a combination of immunohistochemistry (IHC) and in situ hybridization when IHC results were unclear [38]. Epidemiologically, 20–30% of breast cancers overexpress HER2, which is often associated with a worse prognosis. Breast cancer cells may contain about 20–50 copies of HER2 which can be translated into 2 million copies of HER2 at the membrane level [19]. The dependency of breast cancer on HER2 became particularly clear after the clinical implementation of HER2 inhibition with trastuzumab resulted in revolutionary improvement in patient prognosis [39].

Due to its significance, the clinical role and the expression of HER2 has been investigated in a number of other cancers. For instance, about 20% of gastric cancers overexpress HER2, resulting in sensitivity to HER2 inhibitors [40], followed by ovarian cancer (16%), while HER2 overexpression occurs in only 1.3% of colon cancers. In 2024, the FDA approved the use of HER2-targeting ADC trastuzumab deruxtecan for any type of cancer overexpressing HER2 [7]. Thus, even today the expression of HER2 in cancer remains an important and active matter for clinical oncology [41]. Prevalence of HER2 overexpression in different cancers is reported in Table 1. Of note, the numbers vary widely based on the technique/scoring system by which it is assessed, and tumor-specific scoring criteria have often been proposed to improve standardization [40,42]. Importantly, compared to other cancers, including cancers for which HER2 inhibitors are routinely used, USC seems to rank among the highest ones with HER2 overexpression.

### 2.3. HER2 Expression in USC and Its Role in Carcinogenesis

A role of HER2 in USC has been proposed since the early 2000s [74]. Initial studies showed a prevalence of about 14% to 80% of cases with HER2 overexpression [55,56,57,58,59,60,61], accounting for differences in ethnicity [62] and technique [63]. Importantly, as for other cancers, the assessment of the HER2 status in USC has often been the center of debate [75]. HER2 tyrosine kinase receptor overexpression is observed in roughly 35% of women diagnosed with USC, and this gene’s overexpression or amplification occurs more frequently in African American patients compared to Caucasian patients [42,62,76]. Similarly to gastric cancer, USC often exhibits basolateral IHC staining of HER2, rather than apical, and heterogenous staining is often observed [77,78,79,80,81]. Moreover, it has been reported that, in about 8% of USCs, standard antibodies targeting the extracellular domain of HER2 would fail to detect the otherwise high expression of the active intracellular domain by IHC [82]. While a consensus scoring system is still to be developed [75], currently the CAP templates for gynecological malignancies [83] recommend using the slightly modified system that was used by Fader et al. [84,85] (Table 2), even though a recent study reports a 98% concordance between IHC and FISH in a cohort of 1423 USCs [86] when using the 2018 breast cancer guidelines [87]. Based on these new studies, the actual prevalence of HER2 overexpression in USC appears to be around 20–35% [78,86,88,89]. Nevertheless, there seems to be consensus on the fact that HER2 might confer a worse outcome for patients with USC [59,60,76,89,90,91,92].

In USC, the role of HER2 in carcinogenesis at the cellular and molecular level has so far been modeled after the knowledge gained in breast and other cancers. Preclinical research has focused on testing strategies to effectively treat USC by targeting HER2 and understanding chemoresistance to HER2-based therapies, which will be addressed throughout this article. While this approach is more than reasonable, the specific mechanisms by which HER2 contributes to the aggressive nature of USC needs to be further evaluated to improve the efficacy of HER2-based treatments. Through the Cancer Genome Atlas Group and our own work [5,6], whole-exome sequencing showed HER2 to be amplified in 27% to 44% of cases, respectively, while activating PI3K mutations mostly located in exons 9 and 20 were identified in 16% of USC patients. Preclinical data also shows that PI3K/mTOR inhibitors have significant activity in HER2-positive USC cell lines compared to negative lines, suggesting that HER2 might rely on this pathway to promote cancer progression in USC [93,94,95].

## 3. HER2/neu-Directed Therapies

### 3.1. Trastuzumab and Pertuzumab

Trastuzumab is a recombinant IgG1-kappa humanized monoclonal antibody that specifically targets with high affinity (equilibrium dissociation constant (Kd) = 5 nM) the extracellular domain of the human epidermal growth factor receptor 2 (HER2/neu) [96]. Trastuzumab binds to the extracellular ligand-binding region of the HER2/neu receptor, preventing the cleavage of its external domain and promoting receptor downregulation through antibody-mediated mechanisms, and subsequently inhibits HER2-mediated intracellular signaling cascades [97]. Inhibition of the MAPK and PI3K/AKT pathways plays a critical role in trastuzumab-induced cell cycle arrest [98]. Treatment with trastuzumab induces G1 phase arrest, leading to a corresponding decrease in their proliferation [98]. The arrest of the cell cycle is associated with decreased levels of proteins responsible for sequestering the cyclin-dependent kinase (CDK) inhibitor p27^kip^, such as cyclin D1 [99,100,101]. This reduction permits the release of p27^kip^, which binds to the cyclin E-CDK2 complex in the nucleus and represses cell cycle proliferation from the G1 to S phase [99,100,101].

The mechanisms of action are multiple and not completely understood, and the main ones are the inhibition of the ligand-dependent HER2/HER3 heterodimer formation, HER3 phosphorylation, and ultimately association of the p85 subunit of PI3K to HER3 receptors. Inhibition of PI3K complex formation inhibits AKT phosphorylation [102,103]. Moreover, antibody-dependent-cellular-cytotoxicity (ADCC) is another major mechanism of the antitumor function of trastuzumab, which is mediated by effector immune cells, particularly CD56dimCD16-positive NK cells [104,105]. This effect is mainly due to the activation of natural killer (NK) cells, neutrophils, monocytes, and macrophages expressing the Fc gamma receptor III (CD16), which can be bound by the Fc domain of trastuzumab [106,107]. The destruction of tumor cells leads to increased presentation of tumor antigens within the tumor microenvironment, which in turn promotes the activation of antigen-presenting cells and drives T cell polarization [108]. Tumor cells, whether they exhibit low or high levels of HER2 expression, can be targeted and coated by antibodies, ultimately leading to their destruction [109,110].

Trastuzumab was initially approved by the FDA in September 1998 for the first-line treatment of metastatic breast cancer characterized by HER2/neu overexpression [111]. This approval included its use in combination with paclitaxel or as a monotherapy for patients who had previously undergone one or more chemotherapy treatments [112]. Since then, its indications have expanded to include use alongside capecitabine and fluorouracil in the treatment of HER2/neu-positive gastric adenocarcinoma [113]. In the GOG-181B trial, which evaluated trastuzumab as a monotherapy in women with advanced or recurrent endometrial carcinoma, the study failed to meet its target accrual and trastuzumab was considered ineffective for HER2-overexpressing endometrial cancer [114]. However, the final analysis revealed that 47% of patients treated with trastuzumab did not actually exhibit HER2/neu gene amplification in their tumors [114]. Furthermore, the trial imposed no restrictions on the number of prior chemotherapy lines, meaning that many participants had bulky, measurable disease and were heavily pretreated, potentially contributing to the limited observed efficacy [115]. The recommended initial dose of trastuzumab is 4 mg/kg over a 90 min IV infusion, then 2 mg/kg over 30 min by IV infusion weekly. Alternatively, the initial dose can be 8 mg/kg over 90 min IV, then 6 mg/kg over 30–90 min IV every three weeks [116].

In 2018, a randomized Phase II trial of carboplatin–paclitaxel versus carboplatin–paclitaxel–trastuzumab in uterine serous carcinomas with HER2/neu overexpression was conducted and showed that the addition of trastuzumab to carboplatin–paclitaxel was well-tolerated and increased progression-free survival [84]. This study enrolled 61 patients with primary stage III or IV or recurrent HER2/neu-positive disease [84]. According to the most recent overall survival analysis, the median progression-free survival (PFS) was 8.0 months in the control group versus 12.9 months in the experimental arm (*p* = 0.005) [117]. Additionally, overall survival (OS) was significantly improved in the experimental group, with a median of 29.6 months versus 24.4 months in the control group (*p* = 0.046) [117].

In early 2025, a retrospective study conducted by a research team in Taiwan evaluated 280 patients with advanced HER2-positive USCs or carcinosarcomas (CS) [118]. The cohort treated with carboplatin–paclitaxel in combination with trastuzumab demonstrated a significantly longer median overall survival compared to those receiving carboplatin–paclitaxel alone, 41.0 months versus 25.2 months (*p* = 0.002) across both histological subtypes [118]. While the survival benefit was particularly pronounced in patients with CS (*p* < 0.001), a meaningful improvement was also observed in the USC subgroup (*p* = 0.04) [118].

One potential strategy to overcome resistance to trastuzumab is using it in combination with pertuzumab. Pertuzumab is a recombinant humanized monoclonal antibody that targets the extracellular dimerization domain (subdomain II) of HER2 [119]. In contrast to trastuzumab, whose primary mechanism of action is thought to involve the recruitment of host immune cells like natural killer cells to initiate antibody-dependent cell-mediated cytotoxicity (ADCC), pertuzumab is believed to exert its effects by blocking a broader range of downstream signaling pathways [106,107]. This is achieved by inhibiting lateral signal transduction, specifically by preventing HER2 dimerization with other HER family receptors [120]. This mechanism disrupts HER2/HER3 signaling as well as HER2/EGFR heterodimer formation, affecting both HER2-overexpressing cells and those with physiologic HER2 expression levels [121]. Moreover, findings indicate that trastuzumab and pertuzumab bind to distinct regions of the extracellular domain of the HER2 receptor [122]. In 2003, preclinical data about the combination of trastuzumab and pertuzumab in HER2 expressing breast cancer cells showed increased apoptosis and cell growth arrest when compared to trastuzumab alone [123]. The recommended initial dose is 840 mg administered as a 60 min IV infusion, followed every 3 weeks thereafter with 420 mg administered as a 30–60 min IV infusion [124].

Approval in breast cancer was granted based on results from the APHINITY trial (NCT01358877), a multicenter, randomized, double-blind, placebo-controlled study involving 4804 patients with HER2-positive early breast cancer who had undergone surgical removal of the primary tumor prior to randomization [125]. Participants were assigned to receive either pertuzumab or placebo, alongside adjuvant trastuzumab and chemotherapy [125]. The study demonstrated that adding pertuzumab to standard adjuvant therapy improved invasive disease-free survival, particularly among patients with node-positive, HER2-positive early-stage breast cancer [125]. Years later, an in vitro study demonstrated that pertuzumab and trastuzumab each induce similarly strong ADCC and complement-dependent cytotoxicity (CDC) in FISH-positive USC cell lines [126]. However, pertuzumab significantly enhances trastuzumab-induced ADCC in USC cells with low HER2 expression [126]. Recently the TAPUR study evaluated the efficacy of pertuzumab plus trastuzumab in heavily pretreated patients with endometrial cancer with ERBB2/3 amplification, overexpression, or mutation [127]. Among the 28 patients, 13 (46%) had papillary serous carcinoma, 5 (18%) had endometrioid adenocarcinoma, 4 (14%) had clear cell carcinoma, 3 (11%) had mixed mullerian tumors, and the remaining 3 (11%) presented with other types of endometrial malignancies [127]. By achieving a median PFS of 16 weeks and a median OS of 61 weeks across all patients, the study demonstrated that the combination of pertuzumab and trastuzumab exhibits antitumor activity in heavily pretreated patients with endometrial cancer harboring ERBB2/3 amplification [127]. Finally, another randomized phase II/III study is evaluating the use of trastuzumab and hyaluronidase-oysk (an enzyme used in combination with pertuzumab and trastuzumab for the subcutaneous treatment of HER2-positive cancers) or a fixed dose combination of pertuzumab, trastuzumab, and hyaluronidase-zzxf in combination with carboplatin–paclitaxel in patients with HER2-amplified USC or carcinosarcoma [128]. The primary endpoint of this phase II study is PFS and the trial is currently open with further data pending [128].

### 3.2. Trastuzumab Emtansine

Ado-Trastuzumab Emtansine (T-DM1; T-MCC-DM1) is an antibody–drug conjugate targeting the HER2/neu receptor and it is composed of trastuzumab and a cytotoxic moiety (DM1, derivative of maytansine) [129]. Maytansine is an organic heterotetracyclic compound and a 19-membered macrocyclic lactam antibiotic, and it acts as a microtubule inhibitor by binding to tubulin at the rhizoxin-binding site [129]. T-DM1 contains, on average, 3.5 molecules of the cytotoxic agent DM1 attached to each trastuzumab molecule (also known as drug-to-antibody ratio or DAR) [130]. The DM1 is linked to trastuzumab through a stable, non-reducible thioether linker, N-succinidimdyl-4-(N-malemidomethyl) cyclohexane-1-carboxylate [130]. Due to its structure, T-DM1 preserves the therapeutic advantage of trastuzumab while being engineered to deliver the cytotoxic agent DM1 directly into HER2-overexpressing tumor cells. In the landmark EMILIA trial, T-DM1 showed strong clinical efficacy in patients with HER2-positive breast cancer that was resistant to trastuzumab, achieving an objective response rate of 43.6% and a median PFS of 9.6 months with a reported rate of adverse events grade 3 or above of 41% [131]. In 2014, a report investigated the activity of T-DM1 against primary HER2 overexpressing uterine serous carcinoma in vitro and in vivo [132]. In this study, T-DM1 demonstrated significantly greater efficacy than trastuzumab alone in suppressing cell proliferation and inducing apoptosis (*p* = 0.004) in HER2-overexpressing USC cells [132]. Notably, in vivo studies using HER2-overexpressing USC xenograft models showed that T-DM1 markedly reduced tumor growth (*p* = 0.04), and mice treated with T-DM1 experienced significantly prolonged survival compared to those receiving trastuzumab alone or the saline control treatment (*p* ≤ 0.001) [132]. Additionally in a case report, T-DM1 demonstrated its effectiveness in a heavily pretreated patient with recurrent USC overexpressing HER2/neu showing complete resolution of a large metastatic, radiation, and chemotherapy resistant tumor and a prolonged systemic control of disease [133]. Moreover, T-DM1 showed great efficacy compared to trastuzumab in inhibiting cell proliferation (*p* < 0.001) and in inducing G2/M phase cell cycle arrest in uterine and ovarian carcinosarcoma cell lines overexpressing HER2 [134]. The same efficacy was reported in vivo with a reduced tumor growth and a significantly longer survival when compared to trastuzumab and vehicle mice [134].

A single-arm, phase II non-randomized clinical trial is currently underway assessing the efficacy of trastuzumab emtansine in patients with solid tumors harboring HER2 amplification or mutation as identified through comprehensive genomic profiling [135]. The trial encompasses a variety of tumor types. According to the most recent update, clinical activity was observed with objective response rates of 19% in the HER2-mutant cohort and 25% in the HER2-amplified cohort [135]. PFS at 6 months was 31% and 56% for the two groups, respectively, while the median overall survival reached 10.9 months for patients with HER2 mutations and 18.2 months for those with HER2 amplification [135]. No dose reductions were required during treatment. The most frequently reported adverse events of any grade included nausea and fatigue (34%), constipation and elevated liver enzymes (28%), and dry mouth (16%) [135].

The recommended dose of T-DM1 is 3.6 mg/kg given as an IV infusion every 3 weeks (21-day cycle) until disease progression or unacceptable toxicity [136]. Management of adverse events may require temporary interruption, dose reduction, or treatment discontinuation [136].

### 3.3. Trastuzumab Deruxtecan

Trastuzumab deruxtecan (T-DXd) is a novel ADC that has emerged as a promising therapeutic strategy for HER2-positive USC [137,138,139]. At the molecular level, T-DXd comprises a humanized anti-HER2 monoclonal antibody (trastuzumab) linked via a cleavable tetrapeptide linker to a topoisomerase I inhibitor payload (DXd) [137,138,139]. The cytotoxic payload is released through cleavage by capthesin B within the tumor microenvironment [137]. This design facilitates the selective delivery of the cytotoxic agent to HER2-overexpressing tumor cells, enabling intracellular release and induction of apoptosis via DNA damage [137,138,139]. Due to its mechanism of action, T-DXd may offer therapeutic advantages, particularly in case of acquired resistance to trastuzumab or disease progression following trastuzumab-based chemotherapy. T-DXd has shown potent antitumor activity in preclinical USC models and selected case reports, demonstrating efficacy both in vitro and in vivo [140,141]. Notably, T-DXd has demonstrated significant cytotoxic effect and bystander killing in both trastuzumab-sensitive and trastuzumab-resistant cell lines, suggesting that it may be effective in USC regardless of the presence of a constitutively active HER2/PI3K signaling pathway [142].

A key distinguishing feature of T-DXd is its high DAR (8:1) and its bystander effect, which enables the cytotoxic payload to penetrate and affect adjacent tumor cells, regardless of HER2 expression homogeneity [23,143]. Trastuzumab deruxtecan received approval from the FDA in 2019, following the results of the DESTINY-Breast04 trial, which demonstrated its efficacy in HER2-positive breast cancer [144]. Building on this milestone, the STATICE trial, published in March 2023, evaluated the use of trastuzumab deruxtecan in patients with carcinosarcoma and reported significant clinical activity, regardless of HER2 expression levels [145]. More recently in April 2024, the FDA granted tumor-agnostic approval for trastuzumab deruxtecan in the treatment of all unresectable or metastatic solid tumors exhibiting HER2 3+ expression, based on the findings from the DESTINY-PanTumor02 trial [7,146]. In that study, the median PFS was 6.9 months, while the median overall survival (OS) was 13.4 months [146]. The cohort included patients with gynecologic malignancies such as endometrial, cervical, and ovarian cancer, as well as non-gynecologic malignancies like bladder, biliary, pancreatic, and other tumor types [146]. Among 267 patients, 84.6% experienced at least one drug-related adverse event, the most common being nausea (55.1%), anemia (27.7%), and diarrhea (25.8%) [146]. Grade 3 or higher events occurred in 40.8%, with neutropenia and anemia (both 10.9%) being the most frequent [146]. In a separate multicenter randomized trial, the profile of drug-related adverse events was similar, with nausea reported in 72.8% of patients, anemia in 30.4%, and diarrhea in 23.7% [143]. Vomiting was additionally reported in 44% of patients [143]. According to the most recent FDA label, interstitial lung disease (ILD) and pneumonitis have been reported in association with T-DXd. In cases of symptomatic ILD/pneumonitis (Grade 2 or higher), permanent discontinuation of T-DXd and prompt initiation of corticosteroid treatment are recommended [147]. However, the estimated incidence of ILD is approximately 0.5–1%, with most cases being low grade and manageable [148]. With early corticosteroid intervention and the involvement of a multidisciplinary team, treatment can often continue without the need for permanent discontinuation [148].

The recommended dosage of T-DXd in gynecologic malignancies is 5.4 mg/kg given as an IV infusion once every 3 weeks (21-day cycle) until disease progression or unacceptable toxicity. If the dosage needs to be reduced for adverse events, it can be reduced to 4.4 mg/kg, then 3.2 mg/kg before discontinuing the ADC [149]. Currently, there are over 30 ongoing clinical trials registered on clinicaltrials.gov investigating T-DXd for various malignancies. Given the encouraging preclinical and early clinical findings, further investigation of T-DXd and other HER2-targeting therapies is strongly justified in patients with HER2-overexpressing USC, particularly in the recurrent setting following trastuzumab-based treatment.

## 4. Mechanisms of Resistance

The progress made with targeting HER2 in USC has been impacted by the emergence of chemoresistance to HER2 inhibitors, the mechanisms of which are variegated and still not completely understood [150]. As previously explained, HER2 may rely on the activation of the PI3K/mTOR pathway for its oncogenic function in USC [93,94,95]. Preclinical evidence suggests that HER2-positive USC can be upfront resistant to trastuzumab when HER2-overexpression is combined with PI3K oncogenic mutations [151], which are highly prevalent in USC [5,152,153]. Activating mutations in the p110α subunit of PI3K, as well as loss-of-function mutations in PTEN, contribute significantly to trastuzumab resistance by sustaining activation of the PI3K/AKT signaling cascade [154,155,156]. Additionally, certain breast cancer cells showed a truncated form of HER2 that lacks the extracellular domain, thereby preventing trastuzumab from effectively binding to its target [157,158]. Resistance is also promoted by the hyperactivation of alternative tyrosine kinase receptors, such as the insulin-like growth factor 1 receptor (IGF-1R), which can bypass HER2 blockade and maintain downstream signaling despite trastuzumab therapy [159]. However, addition of PI3K or mTOR inhibitors could represent a viable strategy to overcome resistance to HER2 inhibitors [160,161].

Second, in vitro HER2 USC cell lines have been noted to shed the extracellular portion of HER2, lowering the efficacy of trastuzumab by reducing the amount of antibody actively binding at the cell membrane level [162]. This additionally happens through poor HER2-T-DM1 complex internalization and HER2-T-DM1 recycling to plasma membrane [130]. Similarly, ex vivo, HER2-positive USC tends to have a high prevalence of the p95 truncated HER2 variant [163], which is usually associated with poor prognosis and intrinsic resistance to trastzumab due to lacking the extracellular domain. While this could represent a mechanism of resistance to trastuzumab, and potentially its ADCs, it must be noted that the effect of TKIs does not depend on the extracellular domain of HER2, and Afatinib is currently under investigation for recurrent/persistent HER2-positive USC (NCT02491099).

Thirdly, resistance to payloads such as emtansine and DXd may occur through situations such as inefficient lysosomal degradation of T-DM1, and drug efflux via MDR1 expression [164].

Last, intratumoral heterogeneity in the expression of this biomarker is a known problem in scoring HER2 expression in USC [77,78,79,80,81]. This issue has been recently associated with lower therapeutic efficacy of trastuzumab and its conjugate emtansine, and worse PFS [81]. Moreover, there is evidence in the literature of two cases of HER2-positive USC in which HER2 positivity was lost at the time of recurrence, despite successful treatment with HER2 inhibitors [81]. This would support the idea that the environmental pressure applied by HER2 inhibitors eventually results in the selection of treatment resistant clones, which are less addicted to the HER2 pathway, a known hallmark feature of cancer [165,166].

## 5. Discussion and Future Perspectives

Uterine serous carcinomas represent a particularly aggressive form of endometrial cancer, highlighted by resistance to treatment and poor prognosis regardless of stage at diagnosis [2]. While endometrial cancers were previously categorized by overarching features as either type 1 or type 2, the advent of accessible genetic testing of tumors has changed our ability to categorize cancers. We have now been able to find targetable mutations and alterations in tumor cell genomes that, based on the TCGA-dictated classification system, are able to improve the prognosis of patients with aggressive cancers [5,167]. It was through this group’s work that we came to understand that USCs have a high prevalence of HER2 overexpression.

HER2 was already utilized as a targetable biomarker in the treatment of HER2 positive breast cancers with great success. While pathologic scoring between breast and endometrial cancers appears to vary, the effectiveness of HER2-targeted treatment in both cancers when HER2 overexpressed is undeniable. In particular, the seminal trials on HER2 inhibition in USC that we have discussed in this paper showed better PFS and OS with similar safety profiles in patients treated with trastuzumab as compared with patients treated with chemotherapy alone [117,168]. More clinically proven, therapeutic options are expected to be available as USC-specific clinical trials are ongoing. Afatinib, a TKI of HER2, is currently under investigation [169], and future clinical trials with trastuzumab-deruxtecan or other ADCs are warranted.

Here, we have discussed the biologic function of HER2 within normal and tumor cells and how targeted treatments using unconjugated antibodies such as pertuzumab, trastuzumab, and ADCs containing trastuzumab function to prevent tumor proliferation and trigger cell death. These mechanisms are important for understanding the potential ways in which resistance can develop against these agents and could possibly be rectified. We have yet to fully elucidate exactly how resistance develops; however, some methods for overcoming the resistance to unconjugated anti-HER2 antibodies have been identified, like using T-DM1 after trastuzumab [131]. Future work should focus on the elucidation of these mechanisms of resistance more clearly and how to overcome resistance through adjunctive treatment or sequential use of ADCs [170].

Within this review we have also discussed trastuzumab-based ADCs such as trastuzumab emtansine and trastuzumab deruxtecan. These ADCs have the benefit of delivering highly cytotoxic chemotherapy agents directly to HER2-overexpressing cells to allow for even more effective tumor growth inhibition and cell cytotoxicity including bystander killing of HER2-low expressing tumor cells [137]. ADCs have shown great promise in clinical trials and T-DXd is now approved for the agnostic treatment of any solid tumor expressing HER2 at the 3+ level [147]. While adverse events are possible with these medications, vigilance in detection and treatment can prevent complications and allow patients to stay on ADCs long term [148]. Ongoing preclinical studies with T-DXd in our lab are currently focusing on its use in patients with HER2 2+ FISH negative uterine cancers in order to validate whether T-DXd can be effective against this additional population of cancer patients.

A future direction of our research in USC includes the development of a HER2 scoring system that identifies as many patients as possible who would benefit from HER2-targeted treatment. With this in mind, we would currently recommend using the scoring system that was used by Fader et al. [84,85] (Table 2), when scoring HER2 in the clinical setting. This choice is mostly dictated by the fact that, to date, this is the only scoring system that has been proven to result therapeutic success in a clinical trial in USC. Accordingly, a paper by Buza et al. has highlighted the heterogenous nature of HER2 expression in USC patients and emphasized the need for pathologists to identify and mark areas with the strongest intensity of HER2 expression identified by IHC in the specimen for subsequent ISH/FISH testing/scoring. This methodology has been shown to maximize the identification of patients who are considered candidates for HER2-directed treatment with trastuzumab [171]. It is the authors’ opinion that these recommendations should be incorporated in future guidelines.

## 6. Conclusions

In our review, we have discussed the biology of HER2 in normal and cancer cells and demonstrated that HER2 is differentially overexpressed in many different human tumors including biologically aggressive USC. We discussed preclinical and clinical work using HER2-targeted treatments including “naked” antibodies (i.e., trastuzumab and pertuzumab) as well as antibody–drug conjugates (i.e., T-DM1 and T-DXd) in USC models and cancer patients. Finally, we reviewed mechanisms of resistance to HER2-targeted drugs in USC patients, some of which may be overcome with the use of ADCs instead of unconjugated monoclonal antibodies. Future research will focus on the development of more effective algorithms for the identification of USC patients benefitting from HER2-targeted treatments as well as strategies to circumvent mechanisms of resistance to HER2-targeted treatments.

## Figures and Tables

**Table 1:** HER2 expression in various cancer types. The prevalence of HER2 overexpression is denoted for various cancer subtypes, with an especially high prevalence noted for USC.

Cancer Origin	Prevalence of HER2 Overexpression
Bladder	8–70% [40,43,44,45,46]
Breast	11–25% [47]
Stomach	7–34% [40]
Colorectal	1–5% [48,49]
Non-Small-Cell Lung Cancer	6–20% [50,51,52,53,54]
USC	14–80% [55,56,57,58,59,60,61,62,63]
Ovary	4–16% [64,65,66,67]
Prostate	1–24% [68,69,70,71,72,73]

**Table 2:** HER2 scoring algorithm in uterine serous carcinoma. Specified by Buza et al., 2021 [85].

IHC	Definition	Dual-Probe FISH	Results
3+	“Intense complete or basolateral/lateral membrane staining in >30% of tumor cells”	N/A	Positive
2+	“Intense complete or basolateral/lateral membrane staining in ≤30%, or weak to moderate in ≥10% of tumor cells”	“HER2/CEP17 ratio ≥ 2”	Positive
		“HER2/CEP17 ratio < 2.0”	Negative
1+	“Faint/barely perceptible, incomplete membrane staining in any proportion, or weak complete in <10% of tumor cells”	N/A	Negative
0	“No staining in tumor cells”	N/A	Negative

## Data Availability

No new data were created or analyzed in this study. Data sharing is not applicable to this article.

## Abbreviations

The following abbreviations are used in this manuscript:

USC	Uterine Serous Carcinoma
ADC	Antibody–Drug Conjugate
TCGA	The Cancer Genome Atlas Network
T-DM1	Trastuzumab Emastine
T-DXd	Trastuzumab Deruxtecan
FDA	US Food and Drug Administration
IHC	Immunohistochemistry
EGFR	Epidermal Growth Factor Receptor
EGF	Epidermal Growth Factor
FISH	Fluorescence In Situ Hybridization
CDK	Cyclin-Dependent Kinase
NK	Natural Killer
PFS	Progression-Free Survival
OS	Overall Survival
CS	Carcinosarcoma
ADCC	Antibody-Dependent Cell-Mediated Cytotoxicity
ILD	Interstitial Lung Disease
IGF-1R	Insulin-like Growth Factor 1 Receptor
